# The Importance of Type D Personality in the Development of Temporomandibular Disorders (TMDs) and Depression in Students during the COVID-19 Pandemic

**DOI:** 10.3390/brainsci12010028

**Published:** 2021-12-27

**Authors:** Magdalena Gębska, Bartosz Dalewski, Łukasz Pałka, Łukasz Kołodziej, Ewa Sobolewska

**Affiliations:** 1 Department of Rehabilitation Musculoskeletal System, Pomeranian Medical University, 70-204 Szczecin, Poland; mgebska@pum.edu.pl (M.G.); lukas@hot.pl (Ł.K.); 2 Department of Dental Prosthetics, Pomeranian Medical University, 70-204 Szczecin, Poland; bartosz.dalewski@pum.edu.pl (B.D.); rpsobolewski@wp.pl (E.S.); 3 Private Dental Practice, 68-200 Zary, Poland

**Keywords:** COVID-19, stomatognathic system, type of personality, type D personality, TMD, temporomandibular joint, orofacial pain, depression, stress

## Abstract

Background: a type D personality is a factor in a person’s susceptibility to general mental stress, especially during the COVID-19 pandemic. Although many studies were conducted on the relationships among stressful situations, an individual’s personality, depression, and the occurrence of various diseases, e.g., cardiovascular disease or cancer, there are no analogous data on people with temporomandibular disorders (TMDs). Aim: the assessment of TMDs and depression symptoms in students with type D personality. Material and Methods: the research was carried out with the participation of 240 physiotherapy students. The study group (G1) consisted of 120 participants with type D personalities, the control group (G2) consisted of the same number of participants, without “stress” personalities. All subjects were assessed for the occurrence of TMD symptoms, as well as for depression and anxiety symptoms, using the Beck Depression Inventory (BDI), based on the proprietary questionnaire. Results: in students with type D personality symptoms, TMDs occurred significantly more often and in greater number (*p* = 0.00) than in those without stress personalities. The exception was the symptom of increased muscle tension, which showed no statistical difference (*p* = 0.22). Among the 240 respondents, depression was found in 128 people (53.3%). In the group of students with type D personalities, depression was significantly more frequent than in the group without type D personalities (*p* = 0.00). In participants with depression, TMD symptoms were more common, i.e., headaches, neck, and shoulder girdle pain, TMJ acoustic symptoms, increased masticatory muscle tension, teeth clenching, and teeth grinding. There was no significant difference between the incidence of depression and TMJ pain and jaw locking. There was a significant interaction between the occurrence of headaches and acoustic symptoms and the occurrence of depression. For headache and depression interactions, the OR was >1; based on the results, we may assume that a headache depends more on the occurrence of depression rather than it being a symptom of a TMJ disorder in people with type D personalities. Conclusion: type D personality and depression may contribute to the development of TMD symptoms.

## 1. Introduction

The COVID-19 pandemic has created an environment in which many factors associated with a deterioration in the quality of life have exacerbated [1,2,3]. This resulted in a disruption of the education system and contributed to social isolation, which negatively influenced the emotional and physical health of students and university graduates [4]. In addition, the inherent stress that accompanies everyday work and private life may contribute to the emergence (or worsening) of somatic diseases, often of a psychological nature [5,6], such as temporomandibular disorders (TMDs) [7,8,9,10].

Schiffman et al. reported that TMDs might affect 5% to 12% of the population [11]. Some research has found a higher incidence, of up to 25%, and 33% to 40% in the general population [12,13,14]. TMDs are considered a “third” type of dental disease (after dental caries and periodontal diseases), and should be considered a disease that affects the whole human population [15]. TMD symptoms are more common in females [10,11,12,15]. The role of the estrogen receptor ESR1 was discussed recently, in terms of painful TMDs prevalent in females [16]. The most commonly cited etiology includes biological, environmental, emotional, social, and cognitive influencing factors [17]. One of the research works showed an association among TMD symptoms and depression, anxiety, and oral habits, especially bruxism and anxiety, in adolescents [18]. Other studies have emphasized that sleep quality and stress levels are two important concomitant factors for TMDs [19,20]. People with temporomandibular joint (TMJ) disorders are more susceptible to experiencing anxiety and depression [14]. Moreover, there were reports linking TMJ/preauricular pain, depression, and anxiety [21,22,23].

Human health (or disease) is evidenced not only by pathogenic factors, but also by a number of individual variables, mechanisms of behavior, interpersonal interactions, and social causes. Personality is one psychological element that impacts the perception of surrounding stimuli, and one’s way of coping with the occurrence of a pathogenic factor [24].

According to Uher, “personality is a complex set of psychological properties that affect the characteristic patterns of human behavior, invariably in time and situation” [25].

A type D personality, often referred to as a ‘stressed personality’ or ‘distressed personality’, was introduced by clinical psychologist Johan Denollet [26,27,28,29]. Negative effectivity and social inhibition are believed to be the two core traits characterizing type D personalities [30,31,32]. Studies show a clear strong relationship between a type D personality and depression, which is estimated to affect an estimated 3.8% of the population, including 5.0% adults and 5.7% adults over the age of 60 [33,34,35,36]. Depression is distinct from common mood swings and brief emotional reactions to everyday difficulties. In particular, in cases of moderate to severe relapses, depression may become a serious medical condition. It can cause significant suffering to the affected patients and their relatives, as well as poor functioning at work, school, and cause severe personal and family issues over time [37].

A type D personality shows some similarities to the two personality dimensions that make up the ‘big five’, namely neuroticism and introversion. This was confirmed in the research by De Fruyt and Denollet; therefore, it can be assumed that a type D personality is the equivalent to neurotic introversion [38]. 

In their research, Barnett and Gotlib demonstrated the relationship between depression and introversion—that this trait is a predisposing factor for the occurrence of this disease [39]. Personality models can be used as an attempt to explain the relationship between neuroticism and depression, for example, the vulnerability model assumes that neuroticism is a trait that predisposes people to develop depressive disorders, i.e., it is a risk factor. The pathoplastic model draws attention to the relationship between personality traits and the course of depression; for example, an increased level of neuroticism may affect the severity of depression and its duration and, thus, may cause a negative prognosis. The complication model suggests that depression can lead to personality changes. Thus, elevated levels of neuroticism are the result of depression. The spectrum model shows the relationship between depression and neuroticism, from normal to severe levels. At the same time, attention is paid to personality traits (in the context of depression) from the point of view of therapy, which may be hampered by such features as neuroticism and negative emotionality [40].

Research conducted in Spain in the early stages of the COVID-19 epidemic showed that younger adults exhibited greater levels of depression and anxiety when compared to older participants [41,42]. Similar results were obtained in research conducted in China [43,44]. Recent research from British showed that mental health deteriorated significantly between 2018 and April 2020, and this effect was strongest among young adults aged 18–24 [45].

The data above underscore the importance of further work, focusing on people in this age range, to better characterize the impact of the ongoing pandemic on the mental health of young people, and to inform about possible intervention strategies.

Therefore, it can be assumed that young people with type D personalities will be more affected by the COVID-19 pandemic, and will experience stronger health consequences than others.

We hypothesized that type D personality traits may promote the development of depressive states and contribute to the development of TMD symptoms during the COVID-19 pandemic.

The aim of this study was to assess the occurrence of TMDs and depression symptoms in students with type D personalities. 

## 2. Materials and Methods

The research questionnaire was carried out from October 2020 to October 2021 among 240 students studying physiotherapy at the Pomeranian Medical University in Szczecin. The study group (G1) consisted of 120 students who were diagnosed with type D personalities based on the DS-14 questionnaire. The control group (G2) was the same number of participants without type D personalities.

Inclusion criteria in the control group were: first, second, third, or fourth year students of physiotherapy; studying in a hybrid system (stationary and remote); stressed personalities diagnosed on the basis of DS14, no history of neurological, mental, or autoimmune diseases; aged from 20 to 28; consented to participate in the study. Exclusion criterion included chronic diseases (including psychosomatic diseases) and pregnancy. The presence of type D personalities in the G1 group was the differentiating factor in the inclusion criteria in the G1 and G2 groups.

### 2.1. Research Tools

(a) Psychological Questionnaire DS14 (type-D scale). It consists of seven tendencies related to experiencing negative emotions and seven tendencies to refrain from expressing these emotions. Classification to type D requires at least 10 points in each of the two dimensions, i.e., negative affectivity (NA) and social inhibition (SI).
(b) Beck Depression Inventory (BDI) consists of 21 questions. Participants could choose one of four answers for each question. Each is assigned a value of 0 to 3 points. Depending on the sum of the points obtained, it is possible to determine the absence of depression (0–9 points), mild depression (16–24 points), moderate depression (16–24 points), and severe depression (from 25 points).
(c) The proprietary questionnaire for assessing the occurrence of TMD symptoms, i.e., headache, neck and shoulder girdle pain, TMJ pain, TMJ acoustic symptoms, increased masticatory muscle tension, jaw locking, teeth clenching and grinding. The reliability and validity of the applied DS14 and BDI tests were assessed, obtaining the Cronbach’s alpha coefficient of 0.604.

### 2.2. Characteristics of the Studied Group

A total of 164 women (68.3%) and 76 men (31.7%) participated in the study. The mean age in the G1 group of 120 students was 20 years of age (SD 2.32). This group consisted of 99 women (82.5%) and 21 men (17.5%). A total of 56% of participants among the G1 group were first year students, 27% second-year, 12% third-year, and 25% fourth year. In the G2 control group of 120 students, the mean age was 22.95 years of age (SD 6.11). The group consisted of 65 women (54.2%) and 55 (45.8%) men. The G2 group included 41% of first-year students, 32% of second-year students, 17% of third-year students, and 30% of fourth-year students. There was no difference in the sex structure and year of study between the groups, *p* = 0.000.

### 2.3. Statistical Analysis

Data are presented as n, the percentage (%) of responses for qualitative variables, and the average +/− standard deviation for quantitative features. The Chi 2 Pearson test was used to compare the relationships between the qualitative variables. Comparisons for quantitative variables were made using the Student’s *t*-test. The relationship between quantitative variables was assessed using the Pearson correlation coefficient. Due to the large number of cases in the study groups, parametric tests were used based on the central limit theorem. For the frequency of the appearance of TMJ symptoms in patients with type D personalities, and the impact of depression on its occurrence, a logistic regression model was built, which included symptoms, age, sex, and the incidence of depression, diagnosed based on the Beck questionnaire. The model is presented as odds ratios with a 95% confidence interval for the univariate model and adjusted ORs for the multivariate model. The analysis was performed using the RStudio package (Boston, MA, USA). *p* values < 0.05 were considered significant.

## 3. Results

Table 1 provides information on the frequency of occurrence of particular TMD symptoms in the G1 and G2 groups. The two groups were statistically compared. 

As shown in Table 1, students with type D personalities experienced significantly more TMD symptoms than those without stress personalities. The exception was the increased muscle tension symptom, which showed no statistical difference (*p* = 0.22). The most frequently reported symptoms in people with G1 were headache, pain in the neck and shoulder girdle, acoustic symptoms of TMJ, tooth clenching, and TMJ pain. The analysis of the OR index showed that in people with type D personalities, there is an increased frequency of TMD symptoms. 

As shown in Table 2, people with type D personalities have more TMD symptoms than people without stress personality types. There was a statistical difference between the groups.

As shown in Table 3, in the group of all respondents, depression was found in 128 of them (53.3%). In the group of students with type D (G1) personalities, depression was significantly more frequent than in the group without type D (G2) personalities. Moderate depression was predominant in the G1 group and severe depression was also reported. People with mild depression predominated in the G2 group and there were no severe depression cases. 

As shown in Table 4, people with depression are more likely to experience TMD symptoms, such as headaches, neck and shoulder girdle pain, TMJ acoustic symptoms, increased masticatory muscle tension, tooth clenching, and teeth grinding. There was no significant difference between the incidence of depression and TMJ pain and jaw locking. 

**Figure 1:**
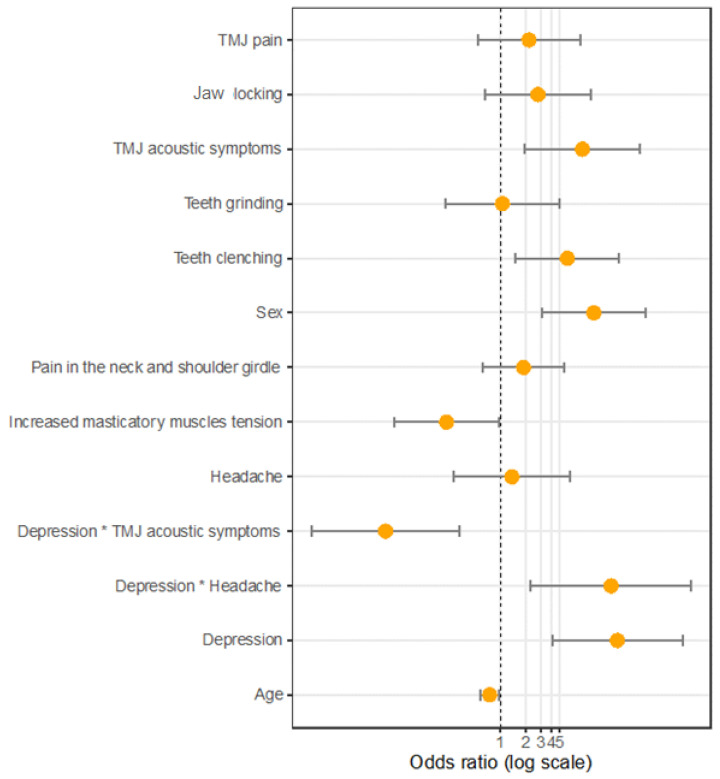
Customized model of the odds ratios.

As shown in Table 5, a significantly more frequent occurrence of acoustic symptoms, increased muscle tension, and tooth clenching was observed in the logistic regression model for the occurrence of the type D personality, including depression. There was a significant interaction between the occurrence of headaches/acoustic symptoms and depression. For the interaction between headaches and depression, the OR was >1; thus, based on the results, we may assume that a headache depends more on the occurrence of depression than it being a symptom of a TMJ disorder in a person with a type D personality (Figure 1).

## 4. Discussion

Personality is one of the most important determinants influencing the behavior and functioning of an individual. Earlier research results confirm the influence of a type D personality on the occurrence of TMD symptoms of TMD during the COVID-19 pandemic [46,47]. According to the present study, people diagnosed with stress personality types reported a greater number of TMD symptoms compared to people without this personality trait (*p* = 0.00). The symptoms most frequently reported in the type D personality group were headache (85.8%), pain in the neck and shoulder girdle (61.7%), tooth clenching (57.5%), TMJ acoustic symptoms (46.7%), TMJ pain (31.7), and teeth grinding (26.7%). All of the above symptoms were significantly more common in students with type D personalities (*p* = 0.00). Thus far, there are no scientific reports in the available literature in which other authors have assessed the above features. Studies conducted in the past twenty years have shown a connection between several psychological variables and TMDs [48,49,50]. It is easy to observe differences in the severity of personality traits, levels of stress, depression, and catastrophic situations between TMD patients and those without the disorder. The insecurity that accompanies long-term suffering is a well-known example of the association between psychosocial risk factors and chronic TMDs [50]. Research conducted by Emodi-Perlaman et al. showed that the COVID-19 pandemic had significant adverse effects concerning the psycho-emotional states of Israeli and Polish populations, causing a worsening of bruxism and TMD symptoms [51]. De Medeiros et al. drew similar conclusions after conducting research on Brazilian medical students. According to the authors, social isolation and stressful situations caused by a pandemic may increase the number of people with TMD symptoms [9]. The research conducted by the authors of the present study shows that TMD symptoms are significant health problems in students, and a type D personality may be considered one of the TMD predictors. 

The COVID-19 pandemic contributed to a severe decline in the general well-being of the global population, increasing the development of depression symptoms [52,53]. The most frequently revealed warning signs of depression are a negative mood, loss of interest, decreased activity, resistance to fatigue, difficulty concentrating, low self-esteem, sleep problems, and pessimism [54]. According to the research conducted by the authors, depression was found in 53.3% of all students. In people with type D personalities, depression was much more frequent (86.7%) than in students without this personality trait (20%) (*p* = 0.00). Particularly noteworthy is the fact that, in students with type D personalities, the most commonly diagnosed degree was moderate (46.7%), and in the control group—slight (16.7%). However, it is disturbing that in people with type D personalities, as many as 20 people (16.7%) were diagnosed with severe depression. According to the research by Gupt et al., conducted on medical students, the occurrence of depression was found in 45.3% of participants [55]. Most of the respondents (34%) had mild depression, while moderate and severe depression were found in 6% and 5.4%, respectively. It was found that 36% of the studied population had type D personalities, along with depression, most of which was of the mild type [55]. According to Sharma, the incidence of depression was 44.5% and it was significantly associated with a type D personality [56]. Similar conclusions were reached by Sensoy et al., who found a strong relationship between type D personalities in students and the occurrence of symptoms of depression (*p* = 0.001) [57]. The above data are very disturbing, as it can be concluded that contemporary academic youth may become victims of depression, which results in lower academic performance, behavioral problems, addiction to drugs, poor socialization and, in extreme situations, suicide attempts. Additionally, depressed people with type D personality traits may experience bad moods, hostility, fear, anger, tension, and perceive themselves in a negative way. 

The comorbidity of depression and numerous pain disorders, including painful TMDs, has already been documented [58,59]. Patients with depression and a comorbid pain disorder have greater pain intensity and functional limitations than those who do not suffer from depression. In addition, pain management is generally less effective in depressed patients. The interaction between pain and depression is likely reciprocal, and there are many theories explaining their relationship. For example, this interaction can be explained by neurobiological factors, as the same neurotransmitters are responsible for transmitting pain and maintaining mood [60]. In addition, TMD patients suffering from stress and depression show increased activity in the masticatory muscles, as well as an increase in the para-functional contacts of the teeth [61,62]. The research by Lajnert et al. in patients with chronic TMDs show that the disease coexists with symptoms of depression and somatization [58]. It can also lower your pain and stress tolerance. Higher levels of depression and somatization were also found in patients with painful TMDs compared to patients with acute TMD symptoms [58]. In studies conducted by Minghelli et al., TMDs were found in 61.4% of students, who developed anxiety and depression (*p* < 0.001) [20]. According to Kindler et al., depression and anxiety symptoms should be taken into account in the diagnosis, prevention, and treatment of TMD pain [22]. 

In our study, we reached similar conclusions. People with depression, compared to healthy controls, more often reported TMD symptoms, i.e., headaches (78.9% vs. 39.3%, *p* = 0.00), pain in the neck and shoulder girdle (57.8% vs. 39.3%, *p* = 0.00), acoustic symptoms of TMJ (40.6% vs. 23.2%, *p* = 0.00), increased masticatory muscle tone (28.9% vs. 17%, *p* = 0.02), tooth clenching (53.9% vs. 17.9%, *p* = 0.00), teeth grinding (26.6% vs. 9%, *p* = 0.00). We did not observe a significant difference in TMJ pain symptoms (*p* = 0.08) and jaw locking (*p* = 0.09). The obtained data may indicate the presence of a chronic state of TMJ disorders, which is less often accompanied by joint pain and more often increased muscle tension, as well as acoustic symptoms and bruxism [63]. This is followed by (usually) long-lasting adaptation of the entire jaw complex and serotonin depletion, resulting in chronic orofacial pain development [64,65]. 

Due to the logistic regression model for the occurrence of TMD symptoms, and a type D personality with depression, carried out by the authors of the study, a significant interaction was observed between the occurrence of a headache and acoustic symptoms and depression. In the analysis, it was observed that headaches occur mainly in people with type D personalities who have been diagnosed with depression, but they do not appear as independent TMD symptoms. Moreover, acoustic symptoms of TMJ in people with type D personalities are more common in the absence of depression. Therefore, we may assume that headaches depend more on the occurrence of depression than being symptoms of TMJ disorders. Moreover, in people with type D personalities, the acoustic symptoms of TMJ present as joint disorders.

The presented results, showing the important role of type D personalities and depression, in the development of TMD symptoms, should be treated with caution, due to the cross-sectional nature of the research and the small group of respondents. It should also be remembered that an individual’s personality is only one of the many factors that determine the formation of TMDs. One significant limitation in the presented research involves the lack of physical examinations of the temporomandibular joints, to objectively assess the presence of dysfunction. Malocclusion, missing teeth, incorrect dental fillings, etc., play significant roles in the formation of TMDs. Moreover, it should be noted that the results obtained in the Beck Depression Inventory (BDI), used for self-assessment of well-being, are only “clues”, not diagnoses of depression. Another significant limitation in the research involves a lack of an analysis of individual factors influencing the perception of the COVID-19 pandemic. Therefore, the authors see the need to continue the research, including the examination of the masticatory organ and the use of more precise psychological tools. 

The authors of this study collected data from the respondents at a specific time, i.e., during the COVID-19 pandemic, which creates an environment of increased stress, uncertainty, and isolation. The results obtained by the authors of this study clearly indicate that TMDs occur more often, and in greater numbers, in people with stressful personality disorders, compared to people without type D personalities. Moreover, people with type D personalities were more likely to suffer from moderate to severe depression than people without type D personalities. Considering all of the above-described limitations of this work, it can be stated—with great caution—that stressful situations (e.g., the COVID-19 pandemic) may contribute to the increase in the number of people (including students) with TMDs, especially people with type D personalities and depression.

Therefore, it is essential to prepare educational systems for young people returning to universities, and to create appropriate support systems in scholar institutions. The primary focus here is on the diagnosis, to reach the most vulnerable young students who may require professional support in mental health, early, and provide subsequent referrals for specialist interdisciplinary help. 

## 5. Conclusions

1. It was found that the occurrences of TMD symptoms are significantly associated with type D personalities and depression in young people.
2. Headaches are related more to an occurrence of depression rather than being symptoms of TMJ disorders in people with type D personalities.
3. This research indicates a high prevalence of type D personalities and depression among students. This means the necessity to implement quick interventions towards diagnosis, treatment, and psychological counselling for academic youth.

## Figures and Tables

**Table 1:** Analysis of the occurrence of TMD symptoms in the study and control groups.

Variable		Type D Personality (G1)	No Type D Personality (G2)	Odds Ratio (or)	95% Confidence Level (95Cl)	Statistical Significance
Headache	yes	103 (85.8%)	42 (35%)	11.25	5.95–21.24	*p* = 0.00
	no	17 (14.2%)	78 (65%)			
Pain in the neck and shoulder girdle	yes	74 (61.7%)	44 (36.7%)	2.77	1.64–4.68	*p* = 0.00
	no	46 (38.3%)	76 (63.3%)			
TMJ pain	yes	38 (31.7%)	17 (14.2%)	2.80	1.47–5.33	*p* = 0.00
	no	82 (68.3%)	103 (85.8%)			
TMJ acoustic symptoms	yes	56 (46.7%)	22 (18.3%)	3.89	2.17–6.99	*p* = 0.00
	no	64 (53.3%)	98 (81.7%)			
Jaw locking	yes	28 (23.3%)	10 (8.3%)	3.34	1.54–7.25	*p* = 0.00
	no	92 (76.7%)	110 (91.7%)			
Increased masticatory muscle tension	yes	32 (26.7%)	24 (20%)	1.45	0.79–2.65	*p* = 0.22
	no	88 (73.3%)	96 (80%)			
Teeth clenching	yes	69 (57.5%)	20 (16.7%)	6.76	3.70–12.34	*p* = 0.00
	no	51 (42.5%)	100 (83.3%)			
Teeth grinding	yes	32 (26.7%)	12 (10.1%)	3.24	1.57–6.66	*p* = 0.00
	no	88 (73.3%)	107 (89.9%)			

**Table 2:** Analysis of the number of TMD symptoms in relation to the presence and absence of the type D personality.

Variable	G1		G2		Statistical Significance
	$\bar{x}$	SD	$\bar{x}$	SD	
Number of symptoms TMDs	3.60	1.50	1.59	1.58	*p* = 0.00

**Table 3:** Analysis of depression occurrences and its severity in the studied group.

Variable	All Subjects (*n* = 240)	G1 (*n* = 120)	G2 (*n* = 120)	Statistical Significance
No depression	112 (46.7%)	16 (13.3%)	96 (80%)	*p* = 0.00
Depression	128 (53.3%)	104 (86.7%)	24 (20%)	
a. Mild depression	48 (20%)	28 (23.3%)	20 (16.7%)	
b. Moderate depression	60 (25%)	56 (46.7%)	4 (3.3%)	
c. Severe depression	20 (8.3%)	20 (16.7%)	0 (0%)	

**Table 4:** Analysis of reported TMD symptoms and the occurrence of depression.

Variable		Depression	No Depression	Statistical Significance
Headache	yes	101 (78.9%)	44 (39.3%)	0 = 0.00
	no	27 (21.1%)	68 (60.7%)	
Pain in the neck and shoulder girdle	yes	74 (57.8%)	44(39.3%)	*p* = 0.00
	no	54 (42.2%)	68 (60.7%)	
TMJ pain	yes	35 (27.3%)	20 (17.9%)	*p* = 0.08
	no	93 (72.7%)	92 (82.1%)	
TMJ acoustic symptoms	yes	52 (40.6%)	26 (23.2%)	*p* = 0.00
	no	76 (59.4%)	86 (76.8%)	
Jaw locking	yes	25 (19.5%)	13 (11.6%)	*p* = 0.09
	no	103 (80.5%)	99 (83%)	
Increased masticatory muscles tension	yes	37 (28.9%)	19 (17%)	*p* = 0.02
	no	91 (71.1%)	93 (83%)	
Teeth clenching	yes	69 (53.9%)	20 (17.9%)	*p* = 0.00
	no	59 (46.1%)	92 (82.1%)	
Teeth grinding	yes	34 (26.6%)	10 (9%)	*p* = 0.00
	no	94 (73.4%)	101 (91%)	

**Table 5:** Logistic regression model for the occurrence of a type D personality, including depression. * the interaction effect between “x” and “y”.

Variable	Type D Personality		
	Statistical Significance	Adjusted OR (aOR)	95% Confidence Level (95%CI)
Sex	0.00	12.49	3.12–49.93
Depression 0/1	0.00	23.54	4.09–135.44
Headache	0.70	1.37	0.28–6.58
Pain in the neck and shoulder girdle	0.26	1.88	0.63–5.58
TMJ pain	0.27	2.18	0.55–8.59
TMJ acoustic symptoms	0.01	9.17	1.95–43.14
Jaw locking	0.16	2.76	0.66–11.52
Increased masticatory muscles tension	0.04	0.23	0.06–0.96
Teeth clenching	0.01	6.10	1.51–24.63
Teeth grinding	0.94	1.06	0.23–4.96
Age	0.03	0.75	0.59–0.97
Depression0/1 * headache	0.01	19.80	2.26–173.71
Depression0/1 * TMJ acoustic symptoms	0.00	0.05	0.01–0.33

## Data Availability

The data presented in this study are available upon request from the corresponding author. The data are not publicly available due to sensitive information.
